# Kallikrein related peptidases 7 and 10 and their substrate desmoglein 3 are upregulated in early stage pancreatic cancerous lesions

**DOI:** 10.1038/s41598-026-48628-7

**Published:** 2026-04-13

**Authors:** Jillian Eisenhauer, Ryan Argo, Alicia K. Fleming Martinez, Emir Maldosevic, Ligia I. Bastea, Cody J. Wehrkamp, Heike R. Döppler, Brandy H. Edenfield, Jason T. Lewis, Michael B. Wallace, Peter Storz

**Affiliations:** 1 Department of Cancer Biology, Mayo Clinic Comprehensive Cancer Center, Mayo Clinic, Jacksonville, FL USA; 2 Department of Laboratory Medicine and Pathology, Mayo Clinic, Jacksonville, FL 32224 USA; 3 Department of Gastroenterology and Hepatology, Mayo Clinic, Jacksonville, FL 32224 USA; 4 Mayo Clinic, 4500 San Pablo Road, Jacksonville, FL 32224 USA

**Keywords:** Pancreatic cancer, PDAC, PanIN lesions, KLK7, KLK10, Desmoglein 3, Biomarkers, Cancer, Cell biology, Gastroenterology, Oncology

## Abstract

Differential expression of Kallikreins (KLKs) was described for established metastatic pancreatic ductal adenocarcinoma (PDAC), but their potential as markers for early detection is not known. We have performed comprehensive in silico and *in situ* analyses of KLK expression in PDAC at different stages of tumor development. We found that upregulation of KLK7 and KLK10 RNA and protein occurs early in tumor development and marks carcinoma in-situ lesions (stage 0, PanIN3) and early-stage (stage 1) PDAC, while non-cancerous low grade lesions stain negative for these proteases. Moreover, both KLKs are co-expressed with desmoglein-3 (DSG3) in PDAC cell lines as well as PDAC samples from treatment naïve patients. DSG3 serves as a substrate for both KLK7 and KLK10 resulting in a 30 kDa extracellular fragment. Overall, our data suggest that analyses for expression of KLK7 and KLK10 as well as their substrates could have potential as diagnostic biomarkers to distinguish non-cancerous low-grade lesions from earliest cancerous lesions in the pancreas.

**Supplementary Information:**

The online version contains supplementary material available at 10.1038/s41598-026-48628-7.

## Introduction

Pancreatic cancer is a devastating disease ranking as the third leading cause of cancer death in the US^1^. The most common form of pancreatic cancer is pancreatic ductal adenocarcinoma (PDAC), which has a 5-year survival rate of 13%^1^. Many PDAC patients are diagnosed after metastasis has occurred, greatly limiting the effectiveness of available treatments. Therefore, to improve patient outcomes, there is a need for detecting pancreatic cancer early in its progression.

Differential expression or activity of proteases can be utilized for PDAC detection. For example, a promising new test (PAC-MANN) is based on a single MMP-sensitive probe that distinguishes PDAC from controls with 79% ± 6% accuracy^2^. Kallikreins (KLKs) are serine proteases that serve regulatory functions throughout the body, including skin desquamation, wound healing, and inflammation, through their ability to break down adherens junction proteins and activate inflammatory cytokines^3–7^. However, overactivation of KLKs has been linked to conditions like Netherton syndrome and psoriasis, as well as a variety of cancers^3–5,7–9^.

KLKs contribute to multiple aspects of tumor development and progression including cell proliferation and survival and pro-angiogenic effects^8^. Through the excessive breakdown of cell adhesion proteins and extracellular matrix (ECM) components, KLKs mediate cancer cell detachment from the tumor and invasion^3–6^. Additionally, KLK activity has been shown to promote epidermal growth factor receptor (EGFR) pathway signaling, which can induce epithelial to mesenchymal transition (EMT) in cancer cells^4,9–11^ but also was linked to earliest events driving the initiation of pancreatic cancer^12^.

KLK substrates include the ECM proteins fibronectin and laminin^13,14^, but also desmogleins, which are involved in maintaining desmosomes to anchor neighboring cells. For example, desmoglein 1 (DSG1) is a substrate for KLK5 in oral squamous carcinoma^3^, and desmoglein 2 (DSG2) is a substrate for KLK7 in pancreatic cancer cell lines^15^. Resulting loss of desmosomes and cell-cell cohesion allows cancer cells to split from the tumor and migrate throughout the microenvironment, potentially leading to metastasis^3,15^.

Expression of specific KLK family members is relegated to distinct areas of the body, and examination of aberrant expression of particular KLKs can be useful for detection of some cancers^4,9^. For example, KLK3, also known as the prostate-specific antigen (PSA), is used for diagnosis and monitoring of prostate cancer^16^, and its substrate activome has been suggested as an additional biomarker to the PSA blood test^9^. Similarly, KLK6 and KLK7 show promise as markers for early detection of ovarian cancer^17^. For pancreatic cancer co-expression of KLK6 and KLK10 has been suggested as a prognostic factor for patient survival^18^, however, no information is available if KLKs can serve as markers for early detection.

Here, we performed a comprehensive analysis of KLK expression in pancreatic cancer exploring KLK family members that are upregulated at earliest stages of PDAC development. We identify KLK7 and KLK10 as potential early detection markers for PDAC and desmoglein-3 (DSG3) which is co-expressed with both KLKs as a substrate. Overall, presence of KLK7, KLK10 and cleaved DSG3 protein may be indicative for developing PDAC.

## Materials and methods

### Cell lines, antibodies and reagents

HPDE (human pancreatic ductal epithelial) control cells were obtained from Dr. M-S. Tsao (Ontario Cancer Institute, Ontario, Canada) and maintained as described elsewhere^19^. All PDAC cell lines (Panc1, MiaPaca2, BxPC3, AsPC1, L3.6, CFPAC1, Capan1) were from the American Type Culture Collection (ATCC, Manassas, VA) and were maintained as recommended by the ATCC. Cell lines were authenticated via their short tandem repeat (STR) profile. Sources, ordering numbers, and dilutions for Western blotting, immunohistochemistry (IHC) or IF-IHC for all antibodies used are listed in detail in Supplemental Table 1.

### Human pancreatic tissue samples

Fully de-identified archival samples were analyzed under a protocol approved by the Mayo Clinic Institutional Review Board (IRB 18–003533). The investigators did not have access to identifiers or codes linking specimens to individuals. Therefore, this research does not meet the definition of human subject research under 45 CFR 46. For microdissections of lesions or ISH/IHC analyses formalin fixed paraffin embedded (FFPE) pancreatic cancer samples from *n* = 10 patients were obtained. For all samples, criteria were that patients had no previous history of chemotherapy treatment, and that LG-PanIN, HG-PanIN and tumors were present in the tissue. Other factors such as patient age, sex or ethnicity were not considered.

### Ethics statement

This study involved the analysis of human specimens that were previously collected at Mayo Clinic Florida. Informed consent was waived by the Mayo Clinic Institutional Review Board due to the use of fully de-identified archival samples. All experiments were performed in accordance with relevant guidelines and regulations.

### Microdissection of human tissues

Human FFPE pancreas samples from patients with no previous history of chemotherapy treatment were used for microdissection. LG-PanIN, HG/PanIN3 lesions and tumor areas were identified by a pathologist (JTL). Approximately 12–15 serial Sect. (10 μm) were cut under RNase free conditions and mounted on PEN-Membrane 2.0 μm slides (Leica, Buffalo Grove, IL) previously cleaned with RNaseZap for 10 min, dried and UV treated overnight. The mounted tissue slides were stained immediately after sectioning according to the Arcturus Paradise PLUS Staining Kit protocol (Thermo Fisher Scientific, Waltham, MA). Immediately after staining, the regions of interest were microdissected using a Leica LMD6500 microscope and collected in the cap of 0.5 ml tubes containing 10 µl of Proteinase K solution (Arcturus Paradise Plus RNA Extraction and Isolation Kit, Thermo Fisher Scientific). The microdissected samples from serial sections of the same patient were pooled and incubated at 37 °C for 16 h. RNA was isolated as described in the Arcturus Paradise Plus RNA Extraction and Isolation Kit (Thermo Fisher Scientific). The RNA quality was analyzed using Agilent RNA 6000 Nano kit (Agilent, Santa Clara, CA) and Agilent Bioanalyzer. The samples were prepped into an Illumina sequencing library using the SMARTer Stranded Total RNA-seq Kit v2 – Pico Input Mammalian Components (TakaraBio, Japan) and sequenced by Azenta Life Sciences (South Plainfield, NJ).

### Immunohistochemistry

Slides were deparaffinized and rehydrated (detailed in^19^). Antigen retrieval was performed with sodium citrate buffer (10 mM, pH 6.0). Then tissue samples were treated with 3% H_2_O_2_ (5 min), washed with 0.5% Tween 20/PBS, and blocked with Protein Block Serum-Free Solution (Agilent, Santa Clara, CA; 5 min, RT). Primary antibodies were diluted in Antibody Diluent Background Reducing Solution (Agilent). Antibodies used and their dilutions are listed in Supplemental Table 1. Staining was visualized using EnVision Plus Anti-Rabbit Labelled Polymer Kit (Agilent), or biotin-streptavidin (Biocare Medical, Concord, CA) 2-step conjugation when primary goat antibodies were used. H&E staining was performed as detailed in^19^. ScanScope XT scanner and ImageScope software (Aperio, Vista, CA) were used to capture images.

### RNAscope *in situ* hybridization (ISH)

*In situ* hybridization (ISH) was performed using RNAscope Assay 2.5 HD Reagent Kit-Brown (Advanced Cell Diagnostics [ACD], Hayward, CA) and the ACD RNAscope Probes Hs-KLK7 (NM_005046.3, target region bp 401–1379, Cat# 478431) and Hs-KLK10 (NM_145888.3, target region bp 770–1787, Cat# 578241). The mRNA signal was detected with DAB, as detailed in^20^. Images were captured using the ScanScope XT scanner and ImageScope software.

### Western blotting

For Western blotting, cells were washed two times with ice-cold PBS (140 mM NaCl, 2.7 mM KCl, 8 mM Na_2_HPO_4_, 1.5 mM KH_2_PO_4_, pH 7.2) and lysed with lysis buffer (50 mM Tris-HCl, pH 7.4, 1% Triton X-100, 150 mM NaCl, 5 mM EDTA, pH 7.4) plus protease inhibitor cocktail (Sigma-Aldrich, St. Louis, MO). Lysates were incubated on ice for 30 min and centrifuged (13,000 rpm, 15 min, 4 °C). After addition of 2x Laemmli buffer, samples were subjected to SDS-PAGE, transferred to nitrocellulose membrane, and proteins of interest were visualized using indicated specific primary antibodies and HRP-conjugated secondary antibodies.

### *In vitro* cleavage (IVC) assays

2 µg purified recombinant 6xHIS-DSG3-ECD (LS-G137004, LS Bio, Seattle, WA) was incubated with either 500 ng purified recombinant KLK7 (ab191666, Abcam, Cambridge, MA) or KLK10 (LS-G38885, LS Bio, Seattle, WA) proteins in 25 µl IVC assay buffer (50 mM Tris, 150 mM -NaCl, pH 8) at 37 °C, for three hours. Reactions were stopped with 2x Laemmli buffer, samples were subjected to SDS-PAGE and Western blotting.

### Data mining

Publicly available RNA-Seq expression data was downloaded from the UCSC Xena platform^21^ for the following datasets: TCGA TARGET GTEx (normal pancreas) and GDC TCGA PAAD (pancreatic tumor).

### Quantification and statistical analysis

All cell biological and biochemical experiments have been performed at least 3 times. In all bar graphs, if not stated otherwise in the figure legends, data are presented as mean ± SD. Box and whisker plots display the median and interquartile range (IQR). Outliers were removed via ROUT (Q = 1%) and p-values were acquired with the unpaired student’s t-test using GraphPad Prism software (GraphPad Inc., La Jolla, CA). *p* < 0.05 was considered statistically significant.

## Results

### Identification of Kallikreins that are indicative for Stage I pancreatic ductal adenocarcinoma (PDAC)

**Fig. 1:**
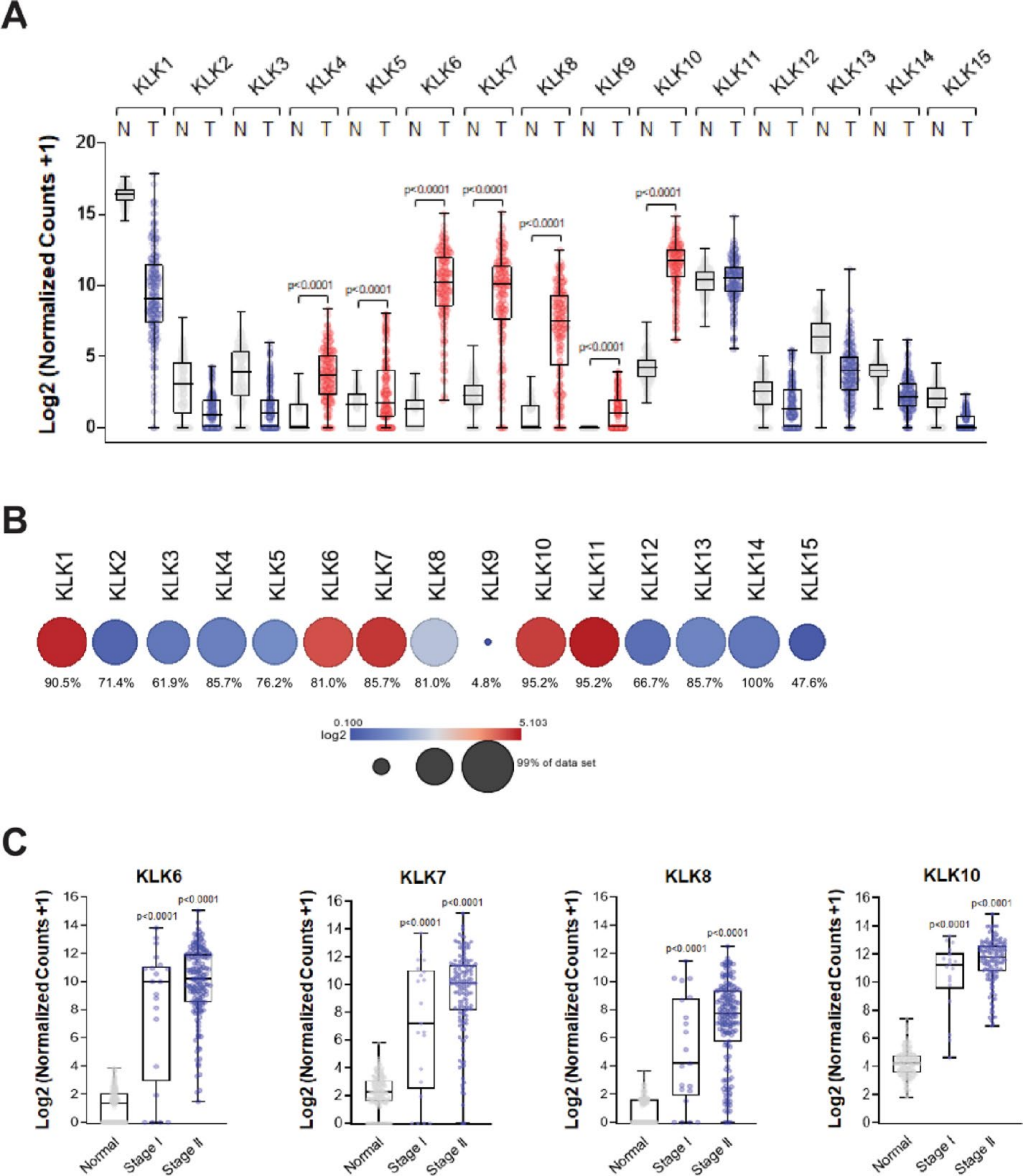
Identification of Kallikreins that are indicative for Stage I pancreatic ductal adenocarcinoma. **A**: Expression (RNA) of KLKs in normal pancreas (N; TCGA TARGET GTEx dataset) and pancreatic tumor (T; GDC TCGA PAAD dataset) tissues. **B**: Bubble graph showing presence of KLKs (RNA) in % of samples of the dataset (mixed pancreatic adenocarcinoma (2022-v32) TCGA-183-tpm-gencode36 dataset) and a cool/warm color scheme indicating level of expression. **C**: RNA expression of indicated KLKs in normal pancreas (TCGA TARGET GTEx) as well as Stage I and II pancreatic cancer (GDC TCGA PAAD).

To identify KLKs that could be indicative for early stages of pancreatic cancer we analyzed publicly available data (UCSC Xena; datasets: TCGA TARGET GTEx for normal; and GDC TCGA PAAD for tumor). We first determined which of the 15 members (KLK1-KLK15) of the KLK protease family are upregulated in PDAC as compared to normal tissue. We found KLK4 to KLK10 expression upregulated in PDAC (Fig. 1A). Of these KLK6, KLK7, KLK8 and KLK10 show a median Log2 (Normalized Counts + 1) higher than 5 (Fig. 1A) and are expressed in more than 80% (KLK6 81%, KLK7 85.7%, KLK8 81%, KLK10 95.2%) of human PDAC samples (Fig. 1B). All are co-expressed in PDAC (Supplemental Fig. S1A), and high expression of KLK6, KLK7, KLK8 and KLK10 decreases overall survival probability of patients (Supplemental Fig. S1B). The separation of samples into Stage I and Stage II PDAC suggested that expression of these four KLKs (6, 7, 8, 10) is already upregulated significantly (*p* < 0.0001) at Stage I (Fig. 1C). KLK4 (expressed in 85.7% of PDA), and KLK9 (expressed in only 4.8% of PDAC), which both show a median Log2 (Normalized Counts + 1) < 5 (Fig. 1A) were also significantly upregulated at Stage I, while KLK5 did not show significant upregulation (Supplemental Fig. S1C). KLK5 (no significance for Stage I) and KLK9 (only 4.8% of patient samples show overexpression) were not further considered.

### Expression of KLK7 and KLK10 distinguishes pancreatic non-cancerous and carcinoma *in situ* lesions

**Fig. 2:**
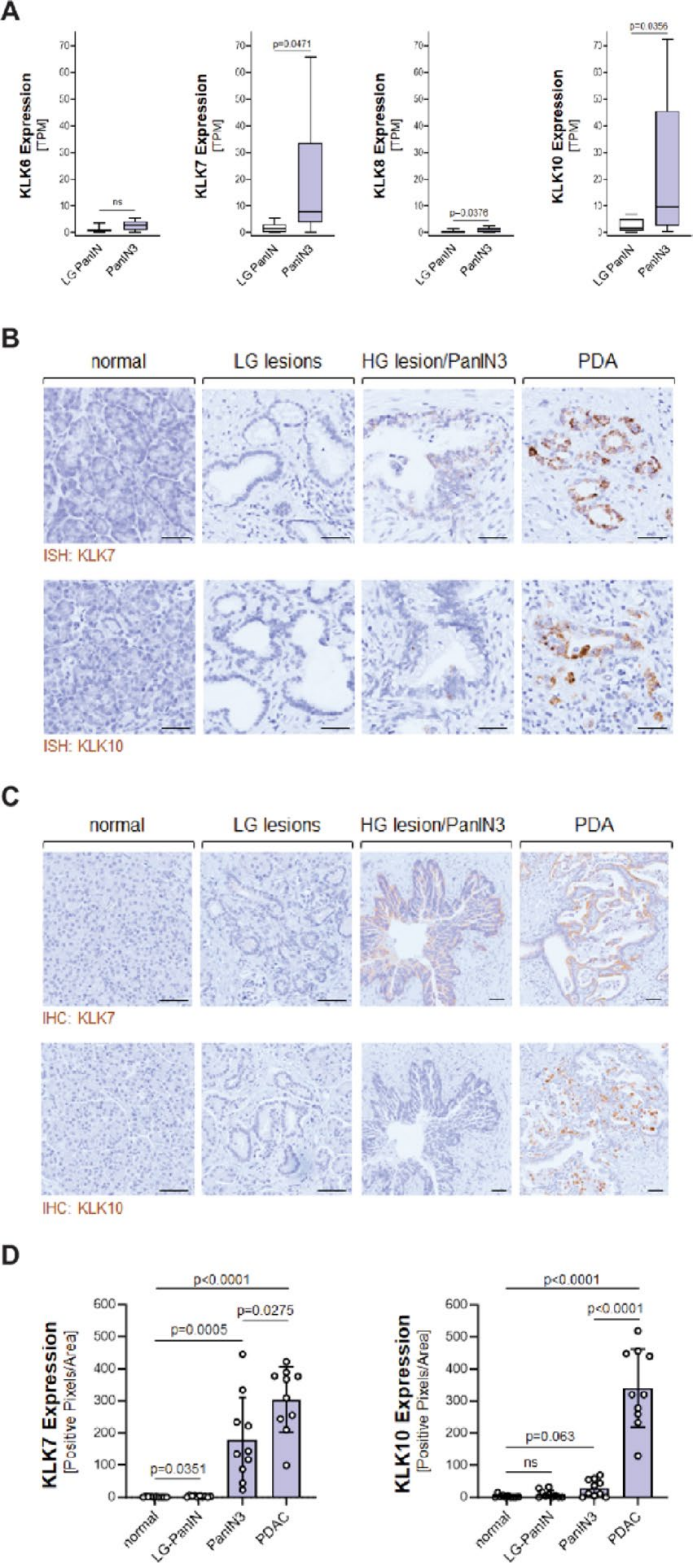
Expression of KLK7 and KLK10 distinguishes pancreatic non-cancerous and carcinoma *in situ* lesions. **A**: RNA expression of indicated KLKs in LG PanIN and HG/PanIN3 lesions microdissected from *n* = 10 patients per group. **B**: *In situ* hybridization for KLK7 or KLK10 mRNA (brown dots) in normal pancreas, LG PanIN, HG/PanIN3 lesions and PDAC areas. The bar indicates 50 μm. **C**: Immunohistochemistry for KLK7 or KLK10 protein (brown staining) in normal pancreas, LG PanIN, HG/PanIN3 lesions and PDAC areas. The bar indicates 50 μm. **D**: Quantification of IHC for KLK7 or KLK10 protein expression in normal pancreas, LG PanIN, HG/PanIN3 lesions and PDAC areas from *n* = 10 patients.

We next determined if KLK4, KLK6, KLK7, KLK8 or KLK10 could be markers for earliest cancerous pancreatic lesions. Therefore, we microdissected pancreatic non-cancerous low-grade (LG) PanIN lesions and cancerous high-grade (HG)/PanIN3 lesions (carcinoma *in situ*) from patient tumors and analyzed them for mRNA expression of above KLKs. While KLK4 expression was not detected in our samples (not shown), KLK6 and KLK8 showed only slight differences in expression between LG-PanIN and HG/PanIN3 lesions (Fig. 2A). In contrast, KLK7 (*p* = 0.0471) and KLK10 (*p* = 0.0356) expression were significantly increased in HG/PanIN3 lesions (Fig. 2A), suggesting that presence of these molecules could allow detection of earliest cancerous lesions. This was confirmed by *in situ* hybridization (ISH). Expression of KLK7 or KLK10 mRNA was detected in HG/PanIN3 lesions and PDAC but not in normal adjacent acinar cell areas or in LG-PanIN lesions, demonstrating that cancerous lesions cells are indeed the producers of KLK7 and KLK10 (Fig. 2B). However, when compared to tumor, RNA abundance for both KLKs was relatively low in HG/PanIN3 lesions. Therefore, we also performed immunohistochemistry for KLK7 or KLK10 and found little to no KLK10 protein present in HG lesions, while KLK7 protein was abundant (Fig. 2C, and quantification in Fig. 2D). This suggests that of all the KLKs, KLK7 is the best candidate to indicate the presence of early pancreatic cancerous lesions in pancreatic tissue.

### Desmoglein 3 is co-expressed with KLK7 in early pancreatic lesions

**Fig. 3:**
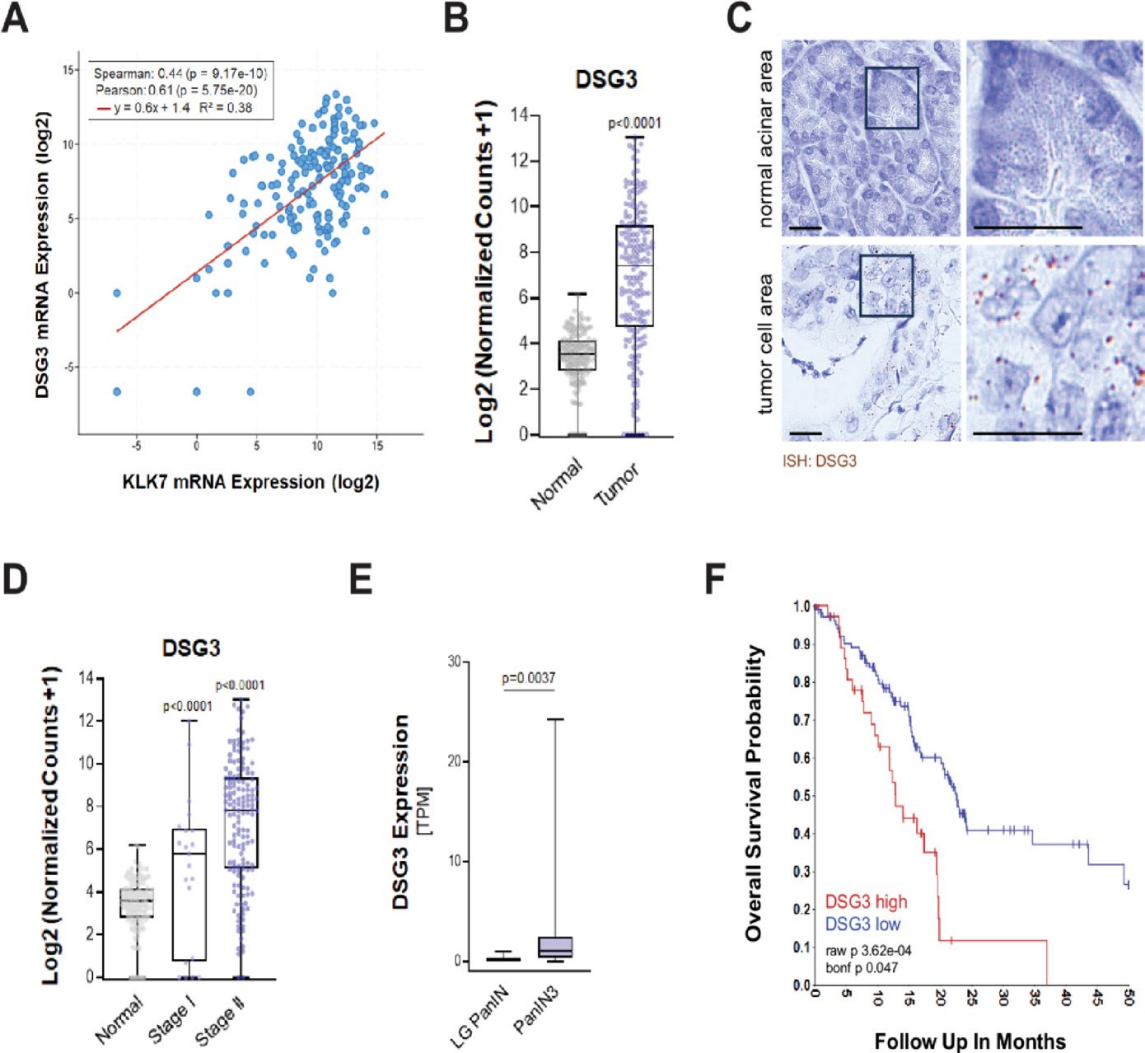
Desmoglein 3 is co-expressed with KLK7 in early pancreatic lesions. **A**: DSG3 and KLK7 mRNA expression data with regression line from https://www.cbioportal.org/ using the TCGA Pancreatic Adenocarcinoma data set (in silico analysis). **B**: RNA expression of DSG3 in normal pancreas TCGA TARGET GTEx data set and pancreatic tumor GDC TCGA PAAD data set (in silico analyses). **C**: *In situ* hybridization for DSG3 mRNA (brown dots) in normal pancreatic acinar and tumor areas. The bar indicates 25 μm. **D**: In silico analyses of RNA expression of DSG3 in normal pancreas (TCGA TARGET GTEx data set) as well as Stage I and II pancreatic cancer (GDC TCGA PAAD data set). **E**: RNA expression of DSG3 in LG PanIN and HG/PanIN3 lesions microdissected from *n* = 10 patients per group. **F**: Overall survival probability of patients either expressing DSG3 at high levels (*n* = 38 samples) or low levels (*n* = 108 samples). Shown is a Kaplan Meyer curve using TCGA data (TCGA-178-rsem-tcgars; DSG3_1830; expression cutoff: 380.6984 (min grp = 8); WITH_SURV (*n* = 146)).

Previous work has shown that KLKs show high abundance in the skin where they modulate the microenvironment through targets that regulate cell adhesion and desmosome-mediated cell-cell contacts^5^. We hypothesized that in PDAC KLK7 and KLK10 may have similar substrates found desmoglein 3 co-expressed with KLK7 in pancreatic tumors (Fig. 3A and Supplemental Fig. S2). Similar as KLK7 and KLK10, DSG3 is significantly upregulated in its expression in PDAC (Fig. 3B), and presence of mRNA in tumor cells was confirmed via in *in situ *hybridization (ISH) on patient tissue (Fig. 3C). To evaluate the value of DSG3 for early detection of PDAC, we determined its RNA expression at Stage I and II tumors and found a significant upregulation at these early tumor stages (Fig. 3D). Moreover, a comparison of DSG3 RNA expression between non-cancerous LG-PanIN lesions and HG/PanIN3 lesions indicated a significant increase in earliest cancerous lesions (Fig. 3E). In accordance with the increased expression of DSG3 at all levels of PDAC development, similar as shown for KLK7 and KLK10 (Supplemental Fig. S1B), the overall survival probability of patients decreases when DSG3 expression levels are high (Fig. 3F).

### Desmoglein 3 is a substrate for KLK7 and KLK10 in PDAC

**Fig. 4:**
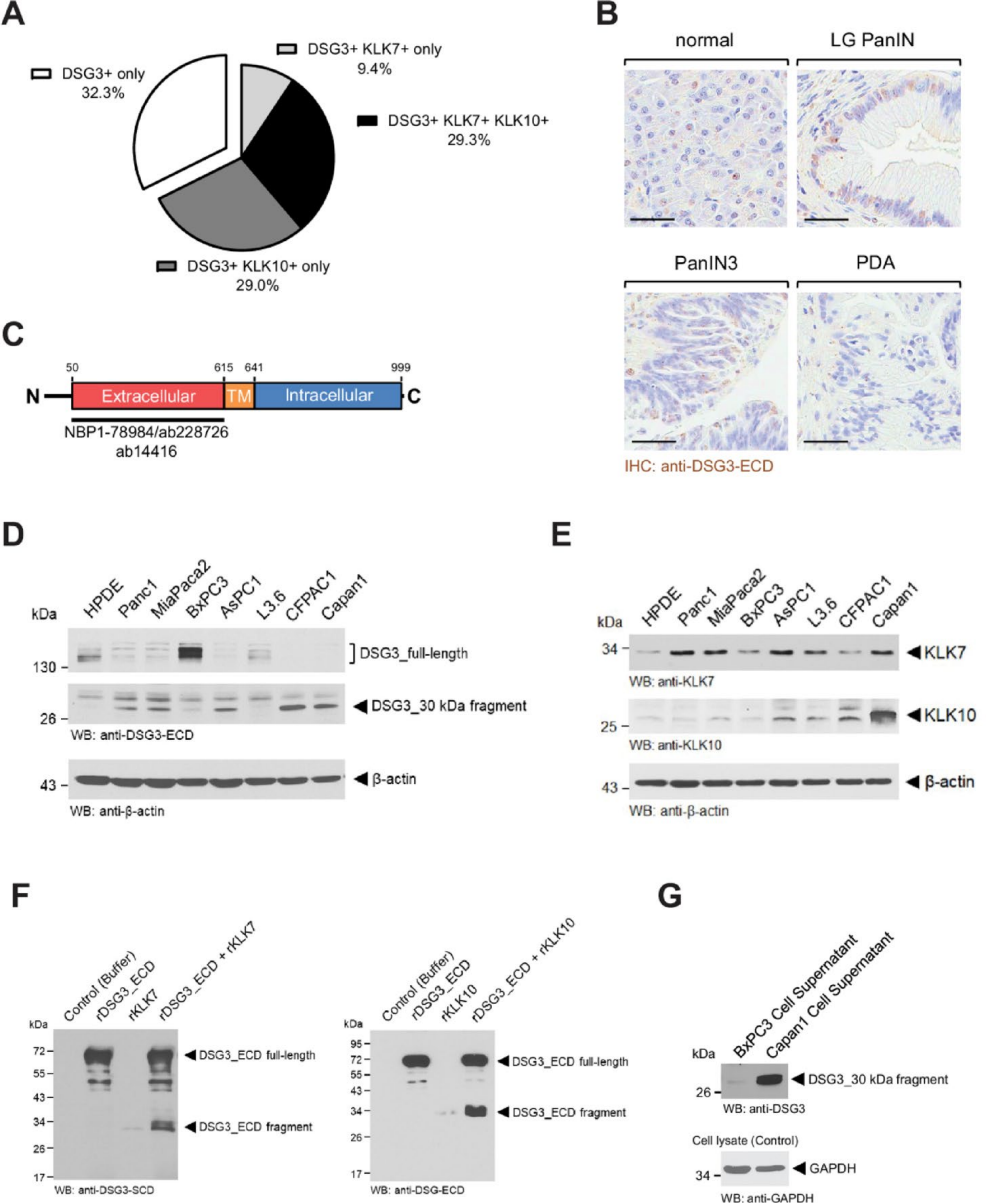
Desmoglein 3 is a substrate for KLK7 and KLK10 in PDAC. **A**: Percentage of DSG3^+^ cells in treatment-naïve PDAC that co-express KLK7, KLK10 or both. In silico analysis of published single-cell RNA expression data from *n* = 11 PDAC patients^22^. **B**: Immunohistochemistry for DSG3 (protein) in normal pancreas, LG PanIN, HG/PanIN3 lesions and PDAC areas. The bar indicates 50 μm. **C**: Scheme of the domains of DSG3 indicating binding sites for the antibodies used. **D**: Cell lysates of HPDE cells or indicated PDAC cell lines were analyzed by Western blot for expression of DSG3. Staining for β-actin served as control for equal loading. **E**: Cell lysates of HPDE cells or indicated PDAC cell lines were analyzed by Western blot for expression of KLK7 and KLK10. Staining for β-actin served as control for equal loading. **F**: *In vitro* cleavage assay with 2 µg recombinant DSG3 and 500 ng KLK7 (left side) or KLK10 (right side). Samples were subjected to SDS-PAGE, transferred to nitrocellulose and visualized via Western blotting for DSG3 (anti-DSG3 antibody). **G**: BxPC3 and Capan1 cells were kept in serum-free media for 48 h. Supernatants and cell lysates were collected. Supernatants were concentrated (Amicon Ultra-4 3 K), subjected to SDS PAGE, transferred to nitrocellulose and analyzed for presence of DSG3. Staining of total cell lysates for GAPDH served as control for equal seeding.

By utilizing a recently published single-cell analyses study^22^ we determined the percentage of DSG3 + treatment naïve tumor cells that also express mRNA for KLK7, KLK10 or both. Overall, 67.7% of samples were positive for DSG3 and either one or both KLKs (Fig. 4A). Based on the analyzed mRNA expression data, we were expecting to detect an increase in DSG3 protein in PDAC, however, immunohistochemistry did not show pronounced differences in DSG3 expression between different lesion stages, and even less presence in PDAC (Fig. 4B, Supplemental Fig. S3A). Since all available antibodies developed for IHC are designed to detect the extracellular domain (ECD) of DSG3 (Fig. 4C), we were speculating that KLK7 and KLK10 may mediate the shedding of the ECD in tumor cells. Analysis of total cell lysates of PDAC tumor cell lines indicated that only BxPC3 and L3.6 cells as well as the human pancreatic ductal epithelial (HPDE) control cell line express full-length DSG3, whereas all other PDAC cell lines showed prominent presence of a DSG3 fragment of 30 kDa that was picked up with antibodies directed against the ECD (Fig. 4D, Supplemental Fig. S5). Interestingly the presence of this fragment correlated with the expression of KLK7 and KLK10 (Fig. 4E) suggesting that DSG3 could be a substrate for these two proteases. This was tested with *in vitro* cleavage assays in which we incubated a recombinant version of the full length ECD of DSG3 with either recombinant KLK7 or KLK10. Both proteases cleaved the full length DSG3 and generated a 30 kDa ECD fragment (Fig. 4F, Supplemental Fig. S5). To demonstrate redundance of both KLKs in cleaving DSG3 in vivo, we utilized BxPC3 cells, which express full-length DSG3 and very low levels of both KLK7 and KLK10. As predicted, ectopic expression of KLK7, KLK10 or both in BxPC3 cells led to occurrence of the 30 kDa DSG3 fragment (Supplemental Fig. S4B, Supplemental Fig. S5). Eventually we analyzed media supernatants from BxPC3 (no DSG3 cleavage) and Capan1 cells (DSG3 cleavage) and found presence of the 30 kDa DSG3 ECD fragment in supernatants from Capan1 cells (Fig. 4G, Supplemental Fig. S5).

## Discussion

More than 66,000 people are diagnosed with pancreatic cancer in the United States annually, and the estimated 5-year survival is about 13%^1^. Diagnosis in over 47% of PDAC patients occurs at a late stage when the tumor has spread to distant organs^1^. At this point, surgery and treatment options for patients are limited and the 5-year survival rate drops to 3%^1,23–25^. On the other hand, the detection of PDAC at Stage I would provide a tremendous survival advantage to the patients, by allowing surgical removal and earlier treatment. For example, catching PDAC at Stage 1 A has been shown to increase 5-year patient survival to over 80%^26^. Ideally tumors are already detected at Stage 0 as carcinoma *in situ* or PanIN3 lesions. At this stage surgery and beginning treatment may be most effective. However, a major barrier to earlier diagnoses and effective surgery or treatment is the lack of early detection methods^27^.

Altered expression of kallikreins has been described for multiple malignant human cancers including breast, colon, ovarian and prostate cancers (summarized in^8^). KLKs are attractive as early detection markers because they are secreted molecules^8^. Moreover, expression of KLK family members changes as different cancers develop^3,7,9–11,28–31^, and detecting these changes shows promise for developing markers for early detection or prognosis^3,18,31,32^. The most prominent example is KLK3, the prostate specific antigen (PSA), which has been established as a blood biomarker for the detection of prostate cancer^16^. For ovarian cancer, KLK6 and KLK7 show promise as markers for early detection^17^.

Here, we performed a comprehensive analysis (in silico) of members of the kallikrein family as markers for established PDAC. While KLK7 and KLK10 overexpression had been described for human PDAC tissue samples, studies did not focus on other KLKs^10,28^. Mass spectrometry of patient samples in recent work identified KLK6 and KLK10 as overexpressed^11^. However, the limitations of all these studies were that they focussed on cell lines and late-stage PDAC samples. This warranted a more detailed analysis of KLK expression indicative for early stages of PDAC development (Stage 1 or Stage 0). Our comprehensive analyses now confirm and expand above findings by showing that KLK6, KLK7, KLK8 and KLK10 are expressed in more than 80% of human PDAC samples and are already significantly upregulated at Stage 1 (Fig. 1). Moreover, by analyzing microdissected lesions, we identify KLK7 as upregulated in earliest cancerous lesions (PanIN3) of the pancreas (Fig. 2), suggesting that presence of KLK7 could be developed as marker for early detection.

Published Gene Ontology (GO) pathway analyses in PDAC patient samples overexpressing KLKs predict increases in activity and expression of genes involved in cell adhesion pathways^11^. In accordance with this, we found the cell adhesion molecule desmoglein-3, involved in maintaining desmosomes to anchor neighboring cells, as upregulated alongside KLK7 at earliest stages of PDAC development and alongside KLK7 and KLK10 in established PDAC (Fig. 3). Analyses of PDAC subtypes indicated significantly increased expression of DSG3 in the basal subtype (Supplemental Fig. S3C), while KLK7 and KLK10 are not differentially expressed between classical and basal PDAC subtypes (Supplemental Fig. S4).

We further show that DSG3 is a substrate of KLK7 (and KLK10) and is cleaved to a 30 kDa extracellular domain fragment that can be detected in the supernatant of cells (Fig. 4). Metastasis happens early in pancreatic cancer progression and is one of the driving forces behind the deadly nature of this disease. Loss of desmosomes and cell-cell cohesion allows cancer cells to split from the tumor and migrate throughout the microenvironment, potentially leading to metastasis^3,15^. Cleavage of DSG3 by KLK7 in early pancreas cancerous lesions may provide an example of how early metastasis may occur.

KLK7 (and KLK10) can also be involved in breaking down additional critical components of the adherens junctions, thus increasing epithelial-to-mesenchymal transition (EMT) and promoting cancer cell detachment from early pancreas cancerous lesions. For example, cleavage of midkine by KLK7 affects the migratory potential of PDAC cells^33^, and KLK7 in PDAC cells lines has shown to promote the shedding of E-cadherin a hallmark of EMT^34^. Similarly, it was shown that aberrant expression of KLK10 promotes PDAC cell metastasis via enhancing EMT^10^. As such it will be interesting to determine in future studies if molecules cleaved by KLK7 and KLK10 can be detected in liquid biopsies of early-stage pancreatic cancer patients. For example, catching PDAC at stage 1 A where it is still confined to the pancreas has been shown to allow surgical removal and increases five-year patient survival rates from 13% to over 80%^26^. It also would allow earlier treatment of patients using natural or synthetic KLK inhibitors^35^. For example, for KLK7, both selective and reversible inhibitors have been described^36,37^.

## Conclusions

In summary, our data indicate that abundance of KLK7, KLK10 and a 30 kDa DSG3 fragment may allow early detection of pancreatic cancerous lesions. A limitation of our study is that this will need rigorous testing in future studies, for example by analyzing pancreatic juice or other less invasive liquid biopsies such as blood plasma for presence of these molecules. Additional future studies are needed to address if above markers can distinguish early stage PDAC from samples from normal patients or patients with chronic pancreatitis (CP). For example, pancreata with CP in addition to inflammation have non-cancerous LG-lesions.

Moreover, additional testing is needed to determine if detection of these molecules may indicate a higher probability of metastasis in patients and may allow predicting overall survival.

## Supplementary Information

Below is the link to the electronic supplementary material.

Supplementary Material 1

## Data Availability

All data generated or analyzed during this study are included in this published article and its supplementary information files. Uncropped Western blots are provided in Supplementary Figure S5. Additional datasets related to this study are available from the corresponding author upon reasonable request.

## References

[1] Siegel, R. L., Kratzer, T. B., Giaquinto, A. N., Sung, H. & Jemal, A. Cancer statistics, 2025. *CA Cancer J. Clin.* **75**, 10–45. 10.3322/caac.21871 (2025). 39817679 10.3322/caac.21871 PMC11745215

[2] Montoya Mira, J. L. et al. Early detection of pancreatic cancer by a high-throughput protease-activated nanosensor assay. *Sci. Transl. Med.* **17**, eadq3110. 10.1126/scitranslmed.adq3110 (2025). 39937880 10.1126/scitranslmed.adq3110

[3] Jiang, R., Shi, Z., Johnson, J. J., Liu, Y. & Stack, M. S. Kallikrein-5 promotes cleavage of desmoglein-1 and loss of cell-cell cohesion in oral squamous cell carcinoma. *J. Biol. Chem.* **286**, 9127–9135. 10.1074/jbc.M110.191361 (2011). 21163944 10.1074/jbc.M110.191361 PMC3059049

[4] Kalinska, M., Meyer-Hoffert, U., Kantyka, T. & Potempa, J. Kallikreins - The melting pot of activity and function. *Biochimie* **122**, 270–282. 10.1016/j.biochi.2015.09.023 (2016). 26408415 10.1016/j.biochi.2015.09.023 PMC4747678

[5] Nauroy, P. & Nystrom, A. Kallikreins: Essential epidermal messengers for regulation of the skin microenvironment during homeostasis, repair and disease. *Matrix Biol. Plus* **6–7**, 100019. 10.1016/j.mbplus.2019.100019 (2020). 33543017 10.1016/j.mbplus.2019.100019 PMC7852331

[6] Sakabe, J. et al. Kallikrein-related peptidase 5 functions in proteolytic processing of profilaggrin in cultured human keratinocytes. *J. Biol. Chem.* **288**, 17179–17189. 10.1074/jbc.M113.476820 (2013). 23629652 10.1074/jbc.M113.476820 PMC3682523

[7] Stefanini, A. C., da Cunha, B. R., Henrique, T. & Tajara, E. H. Involvement of kallikrein-related peptidases in normal and pathologic processes. *Dis. Markers* **2015**, 946572. 10.1155/2015/946572 (2015). 26783378 10.1155/2015/946572 PMC4689925

[8] Borgono, C. A. & Diamandis, E. P. The emerging roles of human tissue kallikreins in cancer. *Nat. Rev. Cancer* **4**, 876–890. 10.1038/nrc1474 (2004). 15516960 10.1038/nrc1474

[9] Lovell, S. et al. A suite of activity-based probes to dissect the KLK activome in drug-resistant prostate cancer. *J. Am. Chem. Soc.* **143**, 8911–8924. 10.1021/jacs.1c03950 (2021). 34085829 10.1021/jacs.1c03950 PMC9282638

[10] Cao, X. Y. et al. Aberrant upregulation of KLK10 promotes metastasis via enhancement of EMT and FAK/SRC/ERK axis in PDAC. *Biochem. Biophys. Res. Commun.* **499**, 584–593. 10.1016/j.bbrc.2018.03.194 (2018). 29621546 10.1016/j.bbrc.2018.03.194

[11] Werner, J. et al. Targeted and explorative profiling of kallikrein proteases and global proteome biology of pancreatic ductal adenocarcinoma, chronic pancreatitis, and normal pancreas highlights disease-specific proteome remodelling. *Neoplasia* **36**, 100871. 10.1016/j.neo.2022.100871 (2023). 36610378 10.1016/j.neo.2022.100871 PMC9841175

[12] Ardito, C. M. et al. EGF receptor is required for KRAS-induced pancreatic tumorigenesis. *Cancer Cell* **22**, 304–317. 10.1016/j.ccr.2012.07.024 (2012). 22975374 10.1016/j.ccr.2012.07.024 PMC3443395

[13] Ramani, V. C. & Haun, R. S. Expression of kallikrein 7 diminishes pancreatic cancer cell adhesion to vitronectin and enhances urokinase-type plasminogen activator receptor shedding. *Pancreas* **37**, 399–404. 10.1097/MPA.0b013e31817f76f7 (2008). 18953252 10.1097/MPA.0b013e31817f76f7

[14] Ramani, V. C. & Haun, R. S. The extracellular matrix protein fibronectin is a substrate for kallikrein 7. *Biochem. Biophys. Res. Commun.* **369**, 1169–1173. 10.1016/j.bbrc.2008.03.021 (2008). 18343220 10.1016/j.bbrc.2008.03.021

[15] Ramani, V. C., Hennings, L. & Haun, R. S. Desmoglein 2 is a substrate of kallikrein 7 in pancreatic cancer. *BMC Cancer* **8**, 373. 10.1186/1471-2407-8-373 (2008). 19091121 10.1186/1471-2407-8-373 PMC2628383

[16] Lilja, H., Ulmert, D. & Vickers, A. J. Prostate-specific antigen and prostate cancer: Prediction, detection and monitoring. *Nat. Rev. Cancer* **8**, 268–278. 10.1038/nrc2351 (2008). 18337732 10.1038/nrc2351

[17] Tamir, A. et al. Kallikrein family proteases KLK6 and KLK7 are potential early detection and diagnostic biomarkers for serous and papillary serous ovarian cancer subtypes. *J. Ovarian Res.* **7**, 109. 10.1186/s13048-014-0109-z (2014). 25477184 10.1186/s13048-014-0109-z PMC4271347

[18] Ruckert, F. et al. Co-expression of KLK6 and KLK10 as prognostic factors for survival in pancreatic ductal adenocarcinoma. *Br. J. Cancer* **99**, 1484–1492. 10.1038/sj.bjc.6604717 (2008). 18854834 10.1038/sj.bjc.6604717 PMC2579692

[19] Bastea, L. I., Hollant, L. M. A., Doppler, H. R., Reid, E. M. & Storz, P. Sangivamycin and its derivatives inhibit Haspin-Histone H3-survivin signaling and induce pancreatic cancer cell death. *Sci. Rep.* **9**, 16588. 10.1038/s41598-019-53223-0 (2019). 31719634 10.1038/s41598-019-53223-0 PMC6851150

[20] Liou, G. Y. et al. The presence of Interleukin-13 at pancreatic ADM/PanIN lesions alters macrophage populations and mediates pancreatic tumorigenesis. *Cell Rep.* **19**, 1322–1333. 10.1016/j.celrep.2017.04.052 (2017). 28514653 10.1016/j.celrep.2017.04.052 PMC5510483

[21] Goldman, M. J. et al. Visualizing and interpreting cancer genomics data via the Xena platform. *Nat. Biotechnol.* **38**, 675–678. 10.1038/s41587-020-0546-8 (2020). 32444850 10.1038/s41587-020-0546-8 PMC7386072

[22] Werba, G. et al. Single-cell RNA sequencing reveals the effects of chemotherapy on human pancreatic adenocarcinoma and its tumor microenvironment. *Nat. Commun.* **14**, 797. 10.1038/s41467-023-36296-4 (2023). 36781852 10.1038/s41467-023-36296-4 PMC9925748

[23] Iacobuzio-Donahue, C. A. Genetic evolution of pancreatic cancer: Lessons learnt from the pancreatic cancer genome sequencing project. *Gut* **61**, 1085–1094. 10.1136/gut.2010.236026 (2012). 21749982 10.1136/gut.2010.236026 PMC3356493

[24] Rhim, A. D. et al. EMT and dissemination precede pancreatic tumor formation. *Cell* **148**, 349–361. 10.1016/j.cell.2011.11.025 (2012). 22265420 10.1016/j.cell.2011.11.025 PMC3266542

[25] Yachida, S. et al. Distant metastasis occurs late during the genetic evolution of pancreatic cancer. *Nature* **467**, 1114–1117. 10.1038/nature09515 (2010). 20981102 10.1038/nature09515 PMC3148940

[26] Blackford, A. L., Canto, M. I., Klein, A. P., Hruban, R. H. & Goggins, M. Recent trends in the incidence and survival of stage 1A pancreatic cancer: A Surveillance, Epidemiology, and End Results analysis. *J. Natl. Cancer Inst.* **112**, 1162–1169. 10.1093/jnci/djaa004 (2020). 31958122 10.1093/jnci/djaa004 PMC7669234

[27] Crawford, H. C., Wallace, M. B. & Storz, P. Early detection and imaging strategies to reveal and target developing pancreatic cancer. *Expert Rev. Anticancer Ther.* **20**, 81–83. 10.1080/14737140.2020.1720654 (2020). 31986932 10.1080/14737140.2020.1720654 PMC7380330

[28] Du, J. P. et al. Kallikrein-related peptidase 7 is a potential target for the treatment of pancreatic cancer. *Oncotarget* **9**, 12894–12906. 10.18632/oncotarget.24132 (2018). 29560118 10.18632/oncotarget.24132 PMC5849182

[29] Emami, N. & Diamandis, E. P. Utility of kallikrein-related peptidases (KLKs) as cancer biomarkers. *Clin. Chem.* **54**, 1600–1607. 10.1373/clinchem.2008.105189 (2008). 18687738 10.1373/clinchem.2008.105189

[30] Shimura, T. et al. Urinary kallikrein 10 predicts the incurability of gastric cancer. *Oncotarget* **8**, 29247–29257. 10.18632/oncotarget.16453 (2017). 28418926 10.18632/oncotarget.16453 PMC5438727

[31] Jiao, X. Overexpression of kallikrein gene 10 is a biomarker for predicting poor prognosis in gastric cancer. *World J. Gastroenterol.* **19**, 9425–9431. 10.3748/wjg.v19.i48.9425 (2013). 24409072 10.3748/wjg.v19.i48.9425 PMC3882418

[32] Petraki, C. et al. Evaluation and prognostic significance of human tissue kallikrein-related peptidase 10 (KLK10) in colorectal cancer. *Tumour Biol.* **33**, 1209–1214. 10.1007/s13277-012-0368-5 (2012). 22437349 10.1007/s13277-012-0368-5

[33] Yu, Y., Prassas, I., Dimitromanolakis, A. & Diamandis, E. P. Novel biological substrates of human kallikrein 7 identified through degradomics. *J. Biol. Chem.* **290**, 17762–17775. 10.1074/jbc.M115.643551 (2015). 26032414 10.1074/jbc.M115.643551 PMC4505025

[34] Johnson, S. K., Ramani, V. C., Hennings, L. & Haun, R. S. Kallikrein 7 enhances pancreatic cancer cell invasion by shedding E-cadherin. *Cancer* **109**, 1811–1820. 10.1002/cncr.22606 (2007). 17354228 10.1002/cncr.22606

[35] Goettig, P., Magdolen, V. & Brandstetter, H. Natural and synthetic inhibitors of kallikrein-related peptidases (KLKs). *Biochimie* **92**, 1546–1567. 10.1016/j.biochi.2010.06.022 (2010). 20615447 10.1016/j.biochi.2010.06.022 PMC3014083

[36] de Veer, S. J. et al. Selective substrates and inhibitors for kallikrein-related peptidase 7 (KLK7) shed light on KLK proteolytic activity in the stratum corneum. *J. Invest. Dermatol.* **137**, 430–439. 10.1016/j.jid.2016.09.017 (2017). 27697464 10.1016/j.jid.2016.09.017

[37] Arama, D. P. et al. Pyrido-imidazodiazepinones as a new class of reversible inhibitors of human kallikrein 7. *Eur. J. Med. Chem.* **93**, 202–213. 10.1016/j.ejmech.2015.02.008 (2015). 25682203 10.1016/j.ejmech.2015.02.008

