# Supplementary material for: Kallikrein related peptidases 7 and 10 and their substrate desmoglein 3 are upregulated in early stage pancreatic cancerous lesions
Source: Sci Rep. 2026 Apr 13;16:17298. doi: 10.1038/s41598-026-48628-7 (PMC13234333; doi:10.1038/s41598-026-48628-7)
Supplement: Supplementary file 1 — Supplementary Material 1 [file 41598_2026_48628_MOESM1_ESM.pdf]

Supplemental Figure S1

A

Correlated Gene	Cytoband	Spearman's Correlation	p-Value	q-Value
KLK8	19q13.41	0.711	6.84E-29	1.36E-24
KLK10	19q13.41	0.651	5.97E-23	5.93E-19
KLK6	19q13.41	0.63	3.75E-21	2.48E-17
KLK9	19q13.41	0.582	1.37E-17	4.52E-14
KLK11	19q13.41	0.535	1.25E-14	2.75E-11

Top 5 KLK genes co-expressed with KLK7

Correlated Gene	Cytoband	Spearman's Correlation	p-Value	q-Value
KLK11	19q13.41	0.742	1.41E-32	2.80E-28
KLK7	19q13.41	0.651	5.97E-23	3.95E-19
KLK8	19q13.41	0.649	8.00E-23	3.97E-19
KLK9	19q13.41	0.638	7.83E-22	2.59E-18
KLK6	19q13.41	0.568	1.08E-16	1.43E-13

Top 5 KLK genes co-expressed with KLK10

B

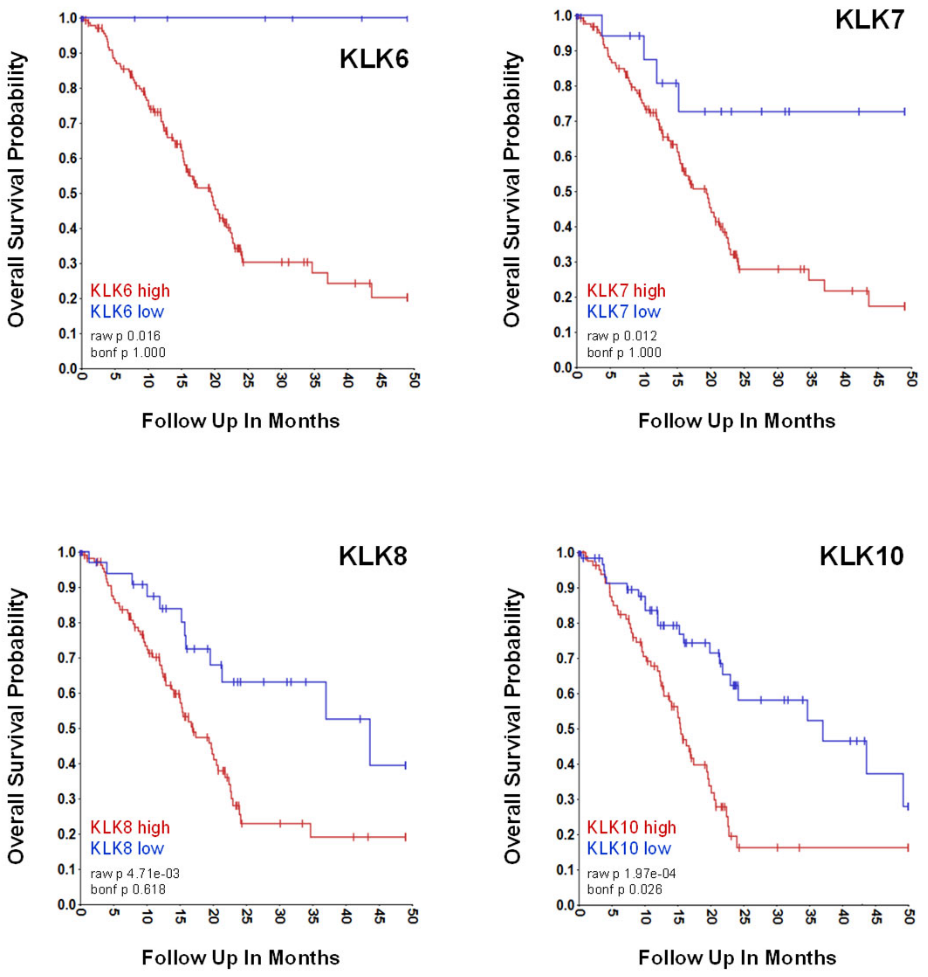

C

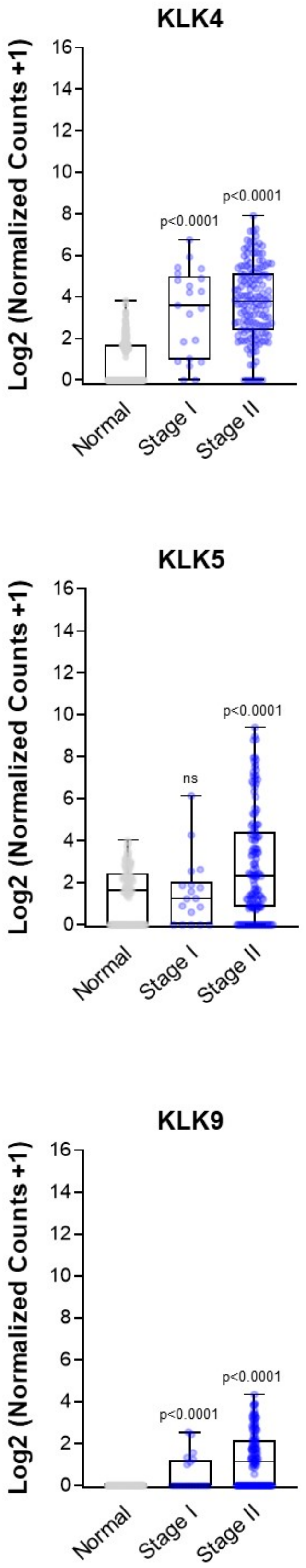

Supplemental Figure S1, supporting Figure 1. **A:** KLKs co-expressing with KLK7 or KLK10 in PDAC. **B:** Overall survival probability of patients expressing indicated KLKs at high or low levels. Shown are Kaplan Meyer Curves using TCGA data. KLK6: TCGA-178-rsem-tcgars; KLK6_5653; expression cutoff: 13.7214 (min grp=8); WITH_SURV (n=146); KLK7: TCGA-178-rsem-tcgars; KLK7_5650; expression cutoff: 77.0282 (min grp=8); WITH_SURV (n=146); KLK8: TCGA-178-rsem-tcgars; KLK8_11202; expression cutoff: 43.1862 (min grp=8); WITH_SURV (n=146); KLK10: TCGA-178-rsem-tcgars; KLK10_5655; expression cutoff: 1841.9642 (min grp=8); WITH_SURV (n=146). **C:** Expression of indicated KLKs in normal pancreas (TCGA TARGET GTEx data base) as well as Stage I and II pancreatic cancer (GDC TCGA PAAD data base).

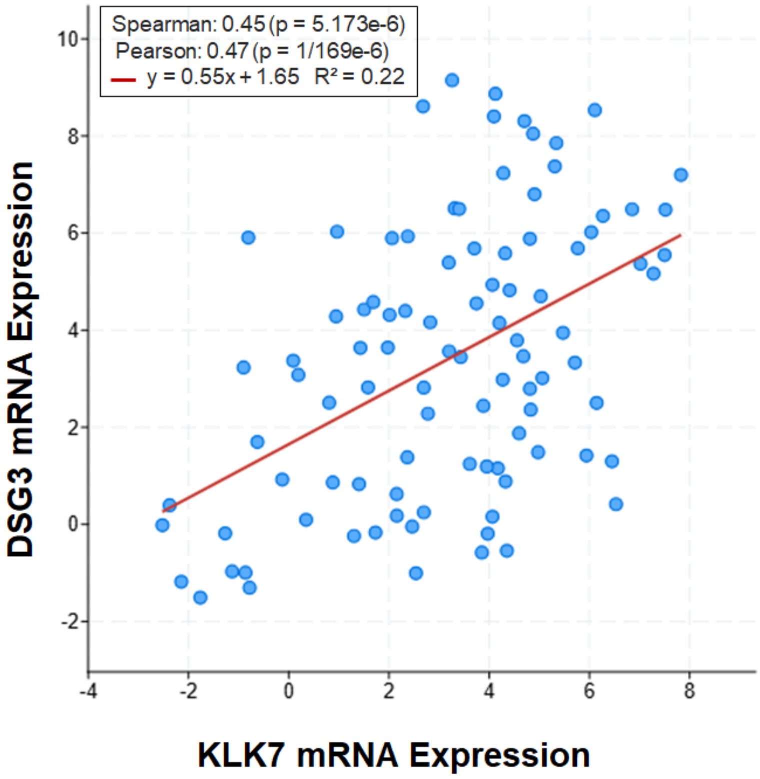

Supplemental Figure S2, supporting Figure 3A. DSG3 and KLK7 mRNA expression data with regression line from *https://www.cbioportal.org/* using the QCMG data set (*in silico* analysis).

Supplemental Figure S3

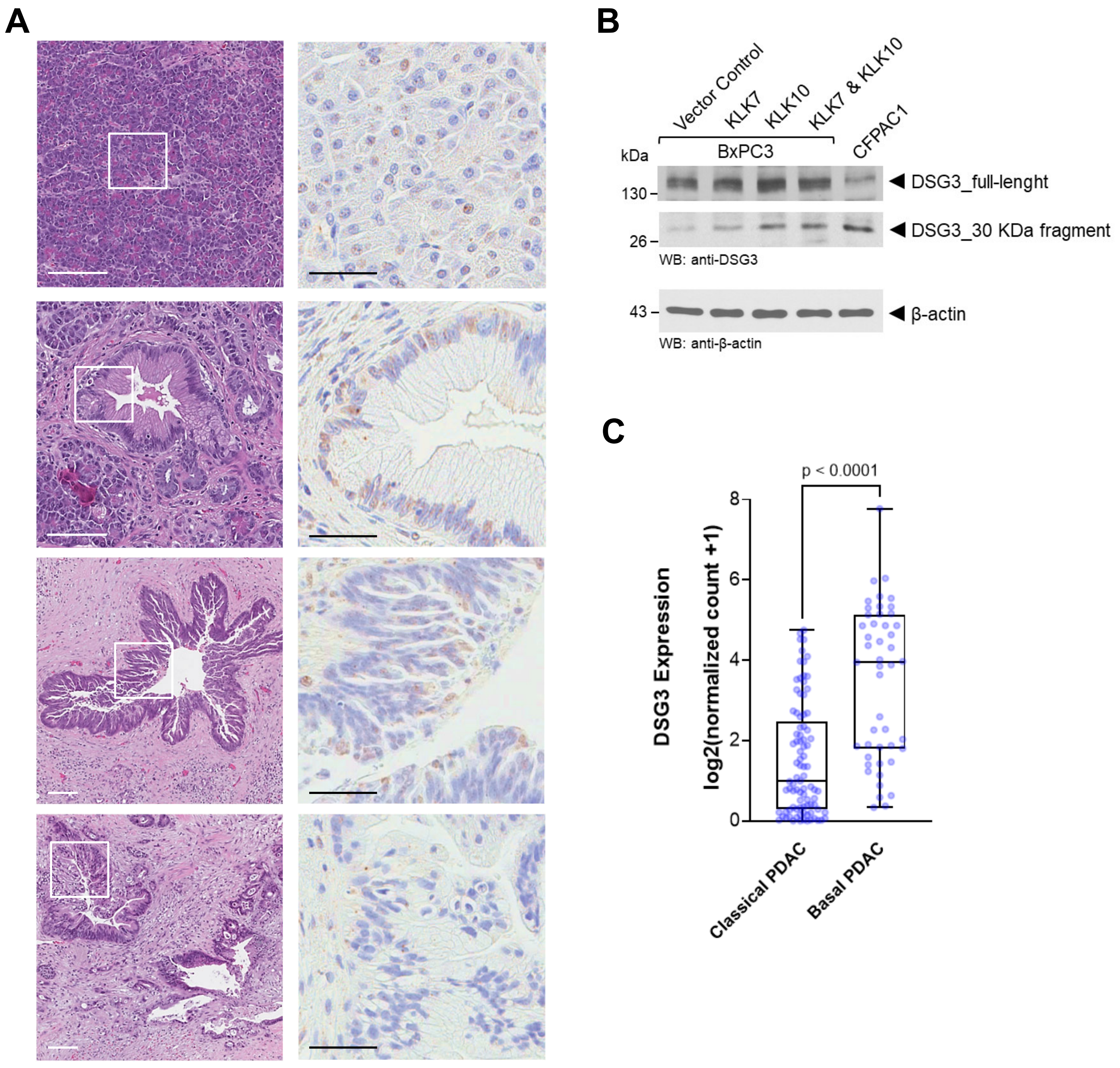

Supplemental Figure S3. **A: Expanded Figure 4B.** Left side: H&E staining accompanying IHC from Figure 4A showing the location of the depicted area within the lesion/tumor. The scale bar indicates 100 µm. Right side: IHC for DSG3 as shown in Figure 4B. The scale bar indicates 50 µm. **B**: BxPC3 cells were transfected either with Vector Control, KLK7, KLK10 or both. Cell lysates were analyzed by Western blot for expression of DSG3 (full-length or 30 kDa fragment). Staining for β-actin served as control for equal loading. CFPAC1 cells (express high level of the 30 kDa fragment) served as positive control. **C**: Expression of DSG3 mRNA in n=165 PDAC (either classical and basal subtype). *In silico* analyses using the GSE224564 dataset.

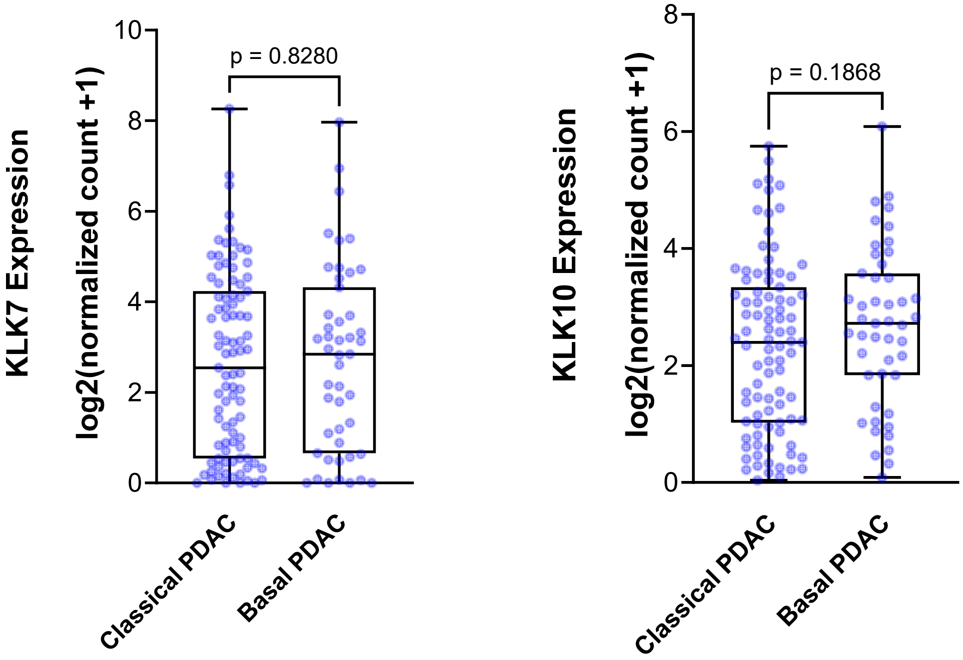

Supplemental Figure S4. Expression of KLK7 (left graph) and KLK10 (right graph) mRNA in n=165 PDAC (either classical and basal subtype). *In silico* analyses using the GSE224564 dataset.

Supplemental Figure S5

Uncropped WBs Fig. 4D:

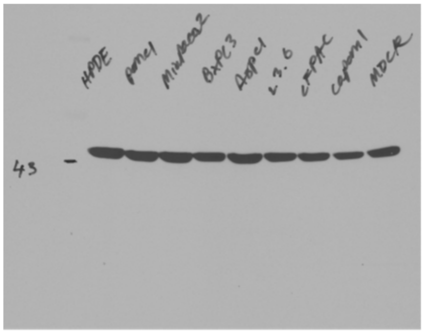

WB: anti-β-actin

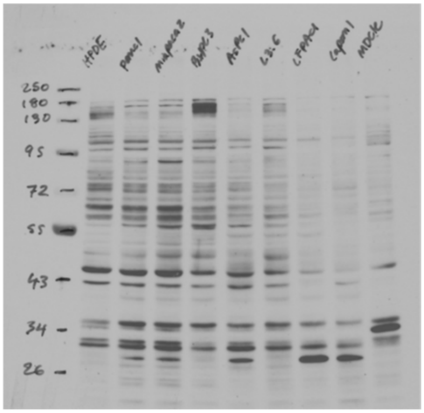

WB: anti-DSG3

Uncropped WBs Fig. 4F:

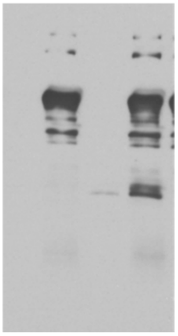

WB: anti-DSG3

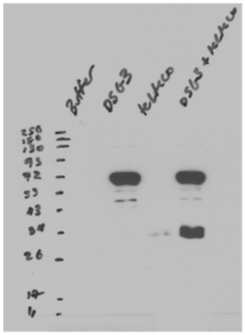

WB: anti-DSG3

Uncropped WBs Fig. 4E:

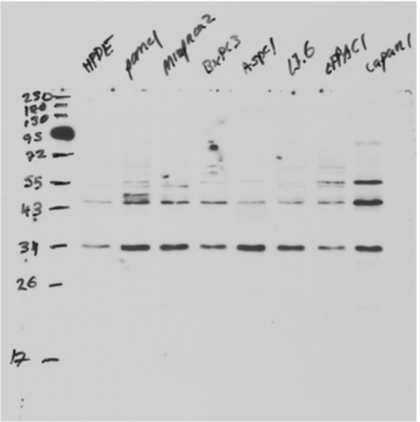

WB: anti-KLK7

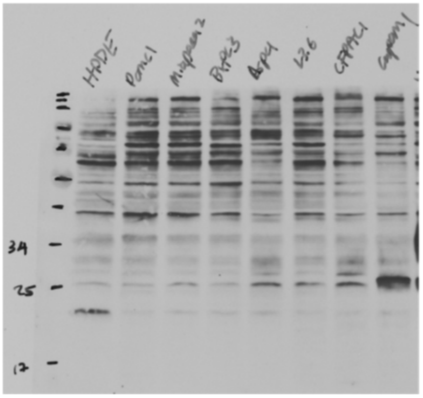

WB: anti-KLK10

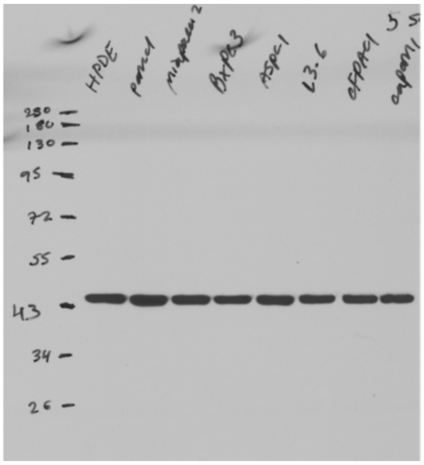

WB: anti-β-actin

Uncropped WBs Fig. 4G:

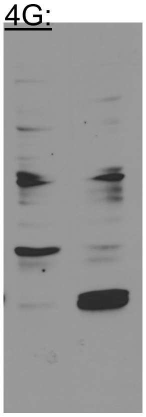

WB: anti-DSG3

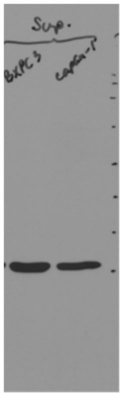

WB: anti-GAPDH

Uncropped WBs Supplemental Fig. S4C:

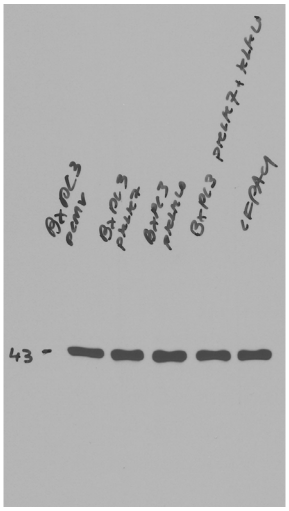

WB: anti-β-actin

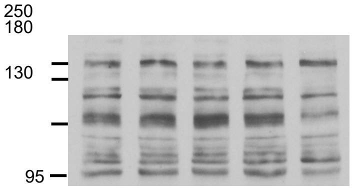

WB: anti-DSG3

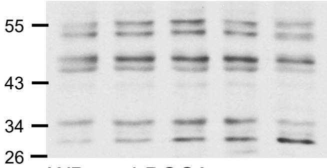

WB: anti-DSG3

Supplemental Table 1

Antibody	Company/Source	Catalog Number	Species	IHC	WB
DSG3	Abcam	ab14416	mouse	1:300	
DSG3	Abcam	ab228726	rabbit		1:1000
DSG3	Novus	NBP1-78984	mouse		1:1000
KLK10	Bioss	bs-2531R	rabbit	1:400	
KLK10	Abcam	ab229968	rabbit		1:1000
KLK7	Abcam	ab96710	rabbit	1:500	1:1000
β-actin	Sigma	A5441	mouse		1:5000

Supplemental Table 1. Antibodies and Dilutions. Antibodies used were from the following sources: Abcam (Cambridge, MA), Novus (Chesterfield, MO), Bioss (Woburn, MA), and Sigma (St. Louis, MO).
